# Therapeutic effects of acylated ghrelin-specific receptor GHS-R1a antagonist in islet transplantation

**DOI:** 10.1038/s41598-021-00740-6

**Published:** 2021-10-28

**Authors:** Kiyoshi Chinen, Naoaki Sakata, Gumpei Yoshimatsu, Masafumi Nakamura, Shohta Kodama

**Affiliations:** 1 Department of Regenerative Medicine and Transplantation, Faculty of Medicine, Fukuoka University, 7-45-1 Nanakuma, Jonan-ku, Fukuoka, 814-0180 Japan; 2 Department of Surgery and Oncology, Graduate School of Medical Sciences, Kyushu University, Fukuoka, Fukuoka 812-8582 Japan

**Keywords:** Endocrinology, Diabetes, Pancreas, Gastrointestinal hormones, Pancreas, Preclinical research, Medical research, Translational research, Cell transplantation

## Abstract

Islet transplantation is a type of cellular replacement therapy for severe diabetes that is limited by compromising effect on engrafted islets. Trials aiming to improve the function of transplanted islets have also been challenging. This study attempted to elucidate whether regulation of growth hormone secretagogue receptor-1a (GHS-R1a), one of the ghrelin receptors, improve the therapeutic effects of islet transplantation using [D-Lys3]-GHRP-6 (DLS), a specific GHS-R1a antagonist. The therapeutic effects of DLS were assessed in terms of the expression/production of endocrine genes/proteins, insulin-releasing function under glucose stimulation of mouse islets, and outcomes of syngeneic murine islet transplantation with systemic DLS administration. DLS treatment promoted insulin production and suppressed somatostatin production, suggesting that cancelation of the binding between ghrelin and GHS-R1a on β or δ cells improved insulin expression. DLS also promoted the glucose-dependent insulin-releasing function of β cells. However, the therapeutic effect of DLS in islet transplantation was fractional. In conclusion, the GHS-R1a antagonist showed preferable effects in improving the therapeutic outcomes of islet transplantation, including the promotion of insulin-releasing function.

## Introduction

Islet transplantation is a promising therapy for patients with severe diabetes mellitus (DM), especially type 1 DM with insulin deficiency. This therapeutic approach enables blood glucose regulation by maintaining appropriate insulin supply from transplanted islets. A recent multicenter phase 3 clinical trial (CIT-07) that enrolled patients with type 1 DM without stimulated C-peptide elevation showed that episodes of severe hypoglycemia proved the usefulness of islet transplantation. Moreover, 87.5% and 71% of the participants achieved improvement in HbA1c level and prevention of severe hypoglycemic events at 1 and 2 years, respectively^1^. Although studies have confirmed the usefulness of this treatment, islet transplantation has still faced some challenges, including limited donor supplies and engraftment difficulty, which may compromise therapeutic outcomes. Regarding limited donor supplies, some novel donor sources, including xenogeneic porcine islets, have been considered a feasible therapeutic option^2^. The development of novel and promising immunosuppressants also contributes toward extending graft survival^3,4^. Furthermore, several preclinical studies on the regulation of innate immunity^5,6^ and ischemia/hypoxia^7,8^ have been promoted, with the aim of improving/supporting the engraftment of transplanted islets. These several trials have sought to improve the *quantity* of engrafted islets. Indeed, clinical islet transplantation data elucidated that multiple transplantations with higher amount of islets contributed to the better outcomes of islet transplantation^9^.

Trials aiming to improve the *quality* of transplanted islets have also been considered pivotal. Moreover, *quality* suggests good insulin-releasing function according to the blood glucose level or toughness against stress caused by the islet isolation and transplantation processes. To establish a novel therapy aiming to improve *quality*, the current study focused on the crosstalk among the islet endocrine cells for ameliorating the insulin-releasing function of β cells, particularly on ghrelin receptors. In general, ghrelin is known as a hunger hormone that induces hyperphagia^10^. Furthermore, this hormone increases blood glucose levels by suppressing insulin release from β cells^11,12^. Ghrelin is classified into two subtypes, namely, acylated and unacylated. Acylated ghrelin binds to a specific receptor named growth hormone secretagogue receptor-1a (GHS-R1a) on β cells and restricts insulin release^13^. This restriction depends on direct and indirect effects via δ cells^10,14,15^. Previous studies have elucidated that acylated ghrelin via GHS-R1a on δ cells induces a decrease in insulin secretion by β cells by promoting somatostatin secretion^15,16^. In contrast, unacylated ghrelin, which does not bind to GHS-R1a, promotes cellular proliferation and increases insulin sensitivity by binding to β cells^17^.

It is considered that specific GHS-R1a suppression prevents the reduction of insulin secretion by maintaining β cell proliferation^10^. However, the usefulness of this treatment in islet transplantation remains unclear. The current study attempted to clarify whether GHS-R1a regulation improved therapeutic effects of islet transplantation.

## Results

### Administration of GHS-R1a antagonist suppressed GHS-R1a expression on islets

Initially, appropriate dose of the GHS-R1a antagonist [D-Lys3]-GHRP-6 (DLS; R&D Systems, Northeast Minneapolis, MN, USA) for administration to isolated islets was determined using the glucose-stimulated insulin secretion (GSIS) assay. Although no difference in the released insulin volume under low-glucose stimulation was observed between the no-treatment and DLS administration groups, a dose-dependent increase in insulin volume under high-glucose stimulation was detected in the DLS group, with the volume being highest at 4.0 µg/mL of DLS (16.73 ± 2.27, 22.41 ± 2.29, 21.93 ± 2.57, and 32.44 ± 1.68 pg/islet × h in the no-treatment, DLS 0.5, 2.0, and 4.0 µg/mL groups, respectively; *p* < 0.01; Supplemental Fig. 1A). Furthermore, administration of 4.0 µg/mL DLS did not impair the viability of islets and residual rate of cultured islets. Regarding viability, approximately 90% of the islets were maintained 7 days after the beginning of the culture in both the no-treatment and DLS 4.0 µg/mL groups (92.17% ± 3.07% in the no-treatment group vs. 87.04% ± 4.42% in DLS 4.0 µg/mL group; Supplemental Fig. 1B, C). Although the residual rates of islets gradually decreased, both groups maintained high levels (79.35% ± 1.94% in the no-treatment group vs. 81.25% ± 2.10% in DLS 4.0 µg/mL group at day 7; Supplemental Fig. 1D). Furthermore, no significant difference in the ratio of Ki-67-positive cells was noted between the no-treatment and DLS groups (2.38% ± 0.21% in the no-treatment group vs. 2.69% ± 0.26% in the DLS group; Supplemental Fig. 2A, B), revealing that DLS treatment did not affect cellular growth of islet cells. Based on the aforementioned data, 4.0 µg/mL was considered an appropriate dose of DLS in this study.

**Figure 1:**
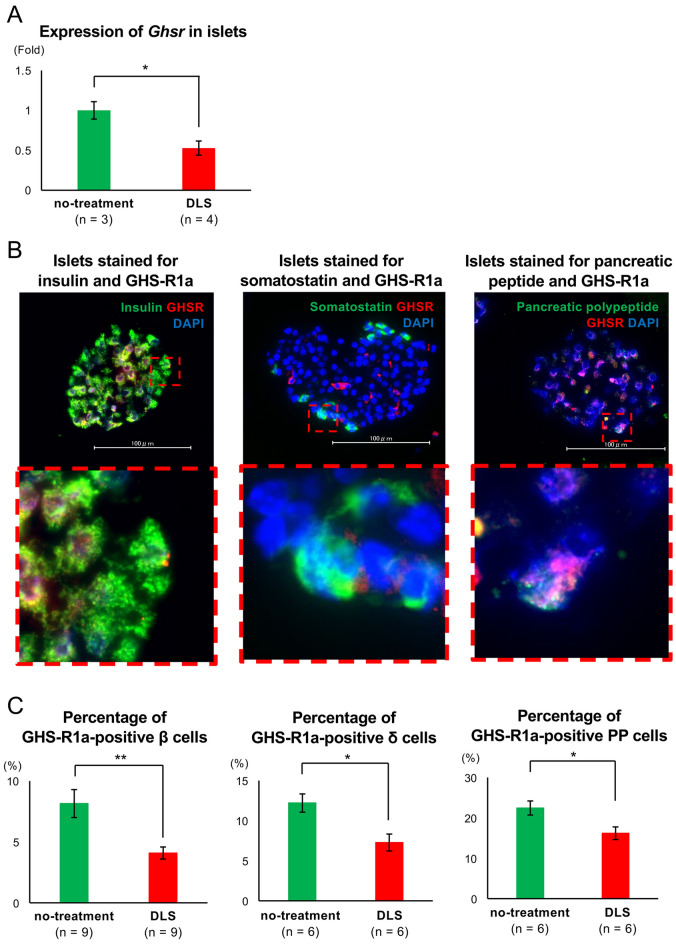
Expression of GHS-R1a on islets. (**A**) Expression of *Ghsr* in islets with and without DLS treatment. Gene expression was quantified using real-time RT-PCR with *Actb* as the internal control. (**B**) Histological findings of DLS-treated islets stained for insulin (*green: left*), somatostatin (*green: center*), pancreatic polypeptide (*green: right*), and GHS-R1a (*red*). Nuclei were stained using DAPI (*blue*). The size of the scale bar is 100 µm. (**C**) Percentage of GHS-R1a-positive β (*left*), δ (*center*), and PP (*right*) cells per each endocrine cells in both no-treatment and DLS groups. Data are presented as means ± SEMs. *P* value of < 0.05 was considered statistically significant; *p < 0.05.

**Figure 2:**
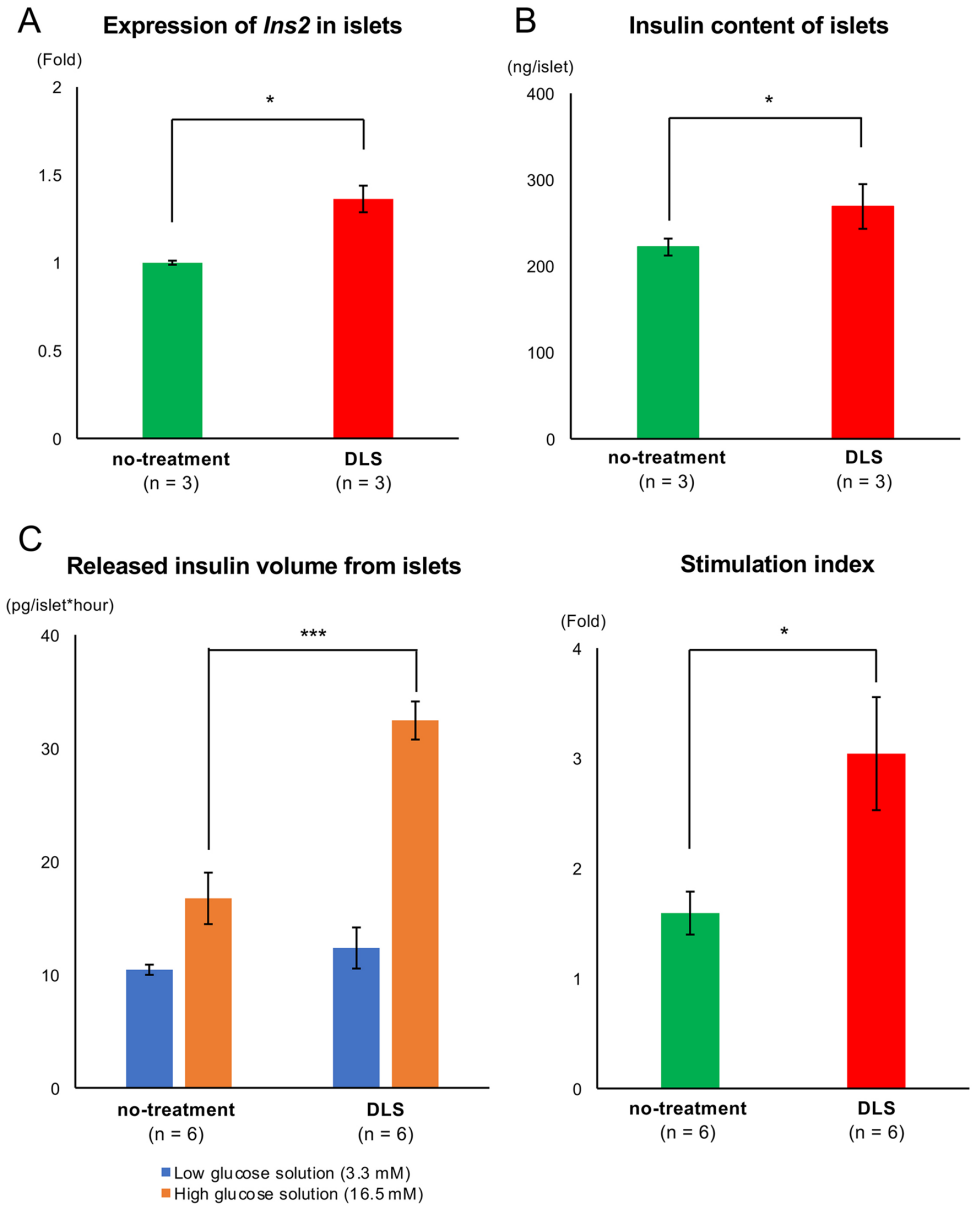
Therapeutic effect of the GHS-R1a antagonist in insulin-releasing function. (**A**) Expression of *Ins2* in islets in the no-treatment and DLS groups. Gene expression was quantified using real-time RT-PCR with *Actb* as the internal control. (**B**) Insulin content of islets in the no-treatment and DLS groups. (**C**) Released insulin volume from islets under low-/high-glucose stimulation (*left*) and stimulation index of islets (*right*) in the no-treatment and DLS groups. Data are presented as means ± SEMs. *P* value of < 0.05 was considered statistically significant; *p < 0.05, ***p < 0.001.

Subsequently, it was assessed whether GHS-R1a expression was detected on islets and whether DLS administration suppressed such expression. Regarding mRNA, *Ghsr* expression in isolated islets was detected via real-time polymerase chain reaction (RT-PCR) and was suppressed by DLS administration (*p* < 0.05; Fig. 1A). Immunohistological examination for isolated islets also elucidated that GHS-R1a expression was detected on insulin-positive cells and that the expression was attenuated by DLS administration. Islets in Fig. 1B were DLS-treated. The percentage of GHS-R1a-positive β cells in the no-treatment group was 8.15% ± 1.15%, which was significantly greater than that in the DLS group (4.09% ± 0.49%, *p* = 0.0074; Fig. 1B, C left). Moreover, some somatostatin-positive cells expressed GHS-R1a on the surface (Fig. 1B, C center). The percentage of GHS-R1a-positive δ cells in the no-treatment and DLS groups was 12.20% ± 1.14% and 7.28% ± 1.05%, respectively (*p* = 0.016; Fig. 1C center). Furthermore, GHS-R1a was expressed on pancreatic polypeptide (PP) cells (Fig. 1B right), the percentage of which (approximately 20%) was higher that on β and δ cells. DLS treatment also decreased the expression of GHS-R1a on PP cells, similar to that on β and δ cells (22.43% ± 1.74% vs. 16.20% ± 1.56%, *p* = 0.035; Fig. 1C right). The aforementioned data indicated that GHS-R1a was expressed on islets, at least on β, δ, and PP cells, with its expression being prominent on PP cells. The administration of GHS-R1a antagonist prevented the expressions.

### Administration of GHS-R1a antagonist improved the insulin-releasing function of islets

The therapeutic impact of DLS on the insulin-releasing function of islets was subsequently examined. DLS treatment promoted the expression of insulin gene *Ins2* (*p* < 0.05; Fig. 2A) and increased internal insulin content in the islets (222.01 ± 4.62 ng/islet in the no-treatment group vs. 269.01 ± 12.19 ng/islet in the DLS group; *p* < 0.05; Fig. 2B). GSIS analysis also revealed that DLS administration increased the volume of insulin released from the islets under high-glucose stimulation (16.73 ± 2.27 pg/islet × h in the no-treatment group vs. 32.44 ± 1.68 pg/islet × h in the DLS group; *p* < 0.001; Fig. 2C), whereas no change was noted under low-glucose stimulation. The DLS-positive group also had a higher stimulation index (i.e., the ratio of released insulin volume between high- and low-glucose solution), comparing with no-treatment group (1.59 ± 0.19 vs. 3.04 ± 0.51; *p* < 0.05; Fig. 2C).

### GHS-R1a antagonist suppressed the expression/production of somatostatin in δ cells

**Figure 3:**
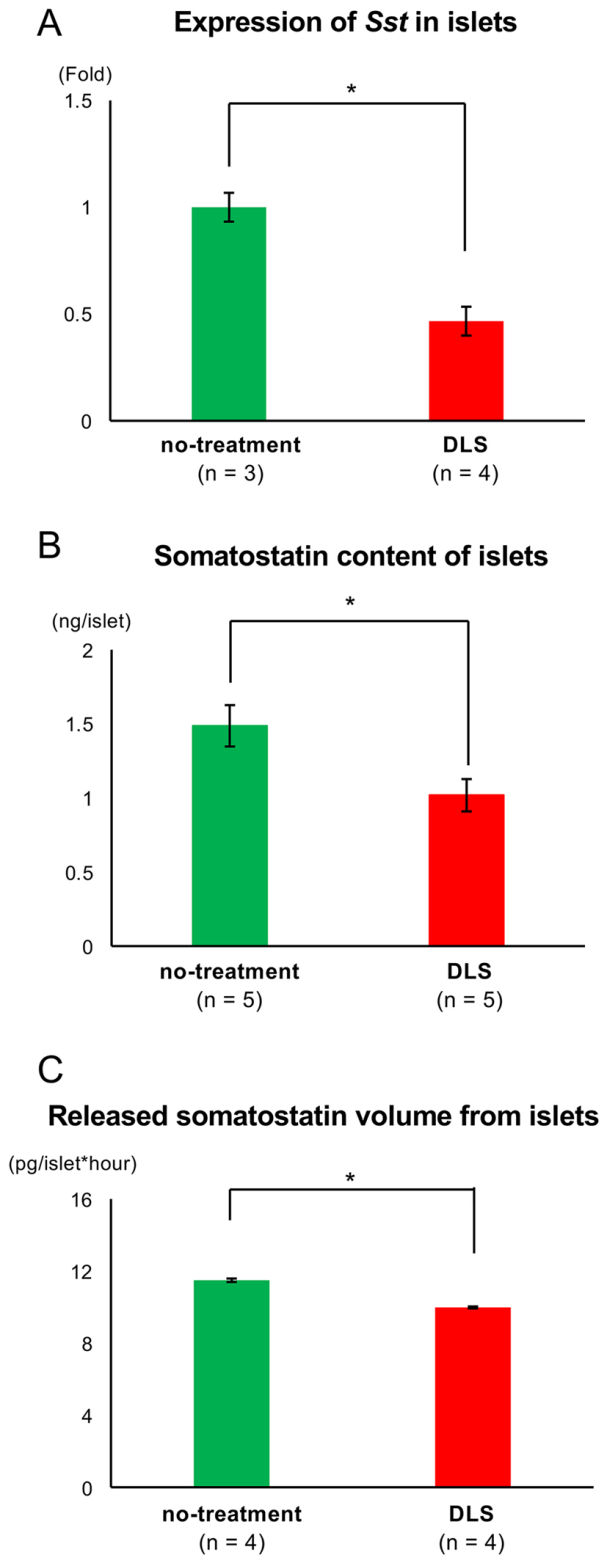
Therapeutic effect of the GHS-R1a antagonist in somatostatin-releasing function. (**A**) Expression of *Sst* in islets in the no-treatment and DLS groups. Gene expression was quantified using real-time RT-PCR with *Actb* as the internal control. (**B**) Somatostatin content of islets in the no-treatment and DLS groups. (**C**) Somatostatin volume released from islets in the culture medium for 24 h. Data are presented as means ± SEMs. *P* value of < 0.05 was considered statistically significant; *p < 0.05.

Figure 3 shows the influence of DLS on the expression/production of somatostatin in δ cells. Notably, DLS treatment significantly suppressed the expression of the somatostatin gene *Sst* (Fig. 3A) but increased the expression of *Ins2* (Fig. 2A). No significant differences in *Gca* and *Ghrl* were observed between the no-treatment and DLS groups (glucagon and ghrelin genes, respectively; Supplemental Fig. 4). DLS also inhibited somatostatin production from the islets (i.e., internal somatostatin content: 1.49 ± 0.14 ng/islet in the no-treatment group vs. 1.02 ± 0.11 ng/islet in the DLS group; *p* < 0.05; Fig. 3B). Furthermore, DLS treatment significantly decreased the volume of somatostatin released from an islet (11.49 ± 0.09 pg/islet × h in the no-treatment group vs. 9.98 ± 0.05 pg/islet × h in the DLS group; *p* < 0.05; Fig. 3C). These findings, including the location of GHS-R1a on endocrine cells (Fig. 1B), indicated that GHS-R1a antagonist inhibited the production/secretion of somatostatin in δ cells and promoted the insulin release from β cells by prohibiting the binding between acylated ghrelin and GHS-R1a.

**Figure 4:**
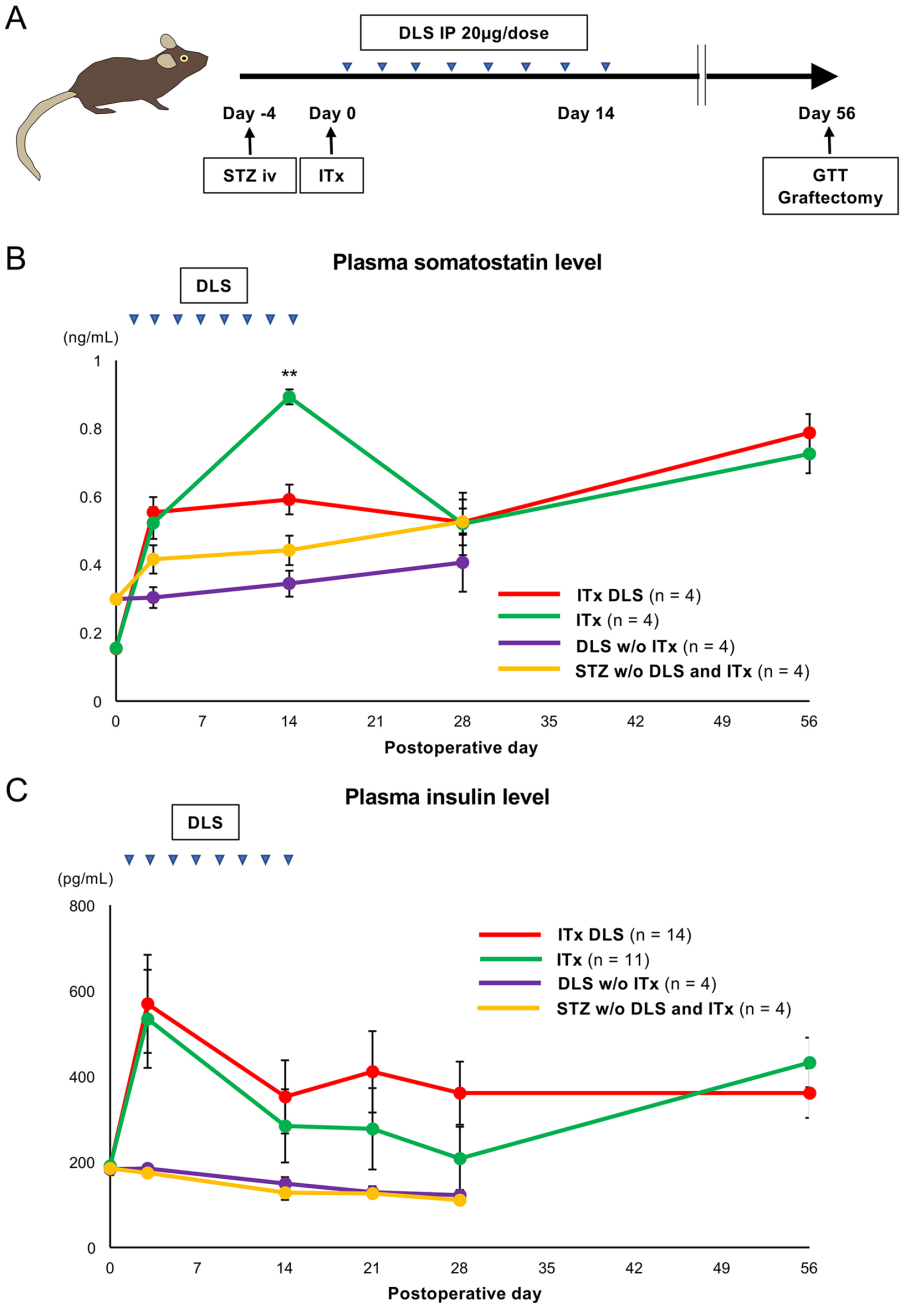

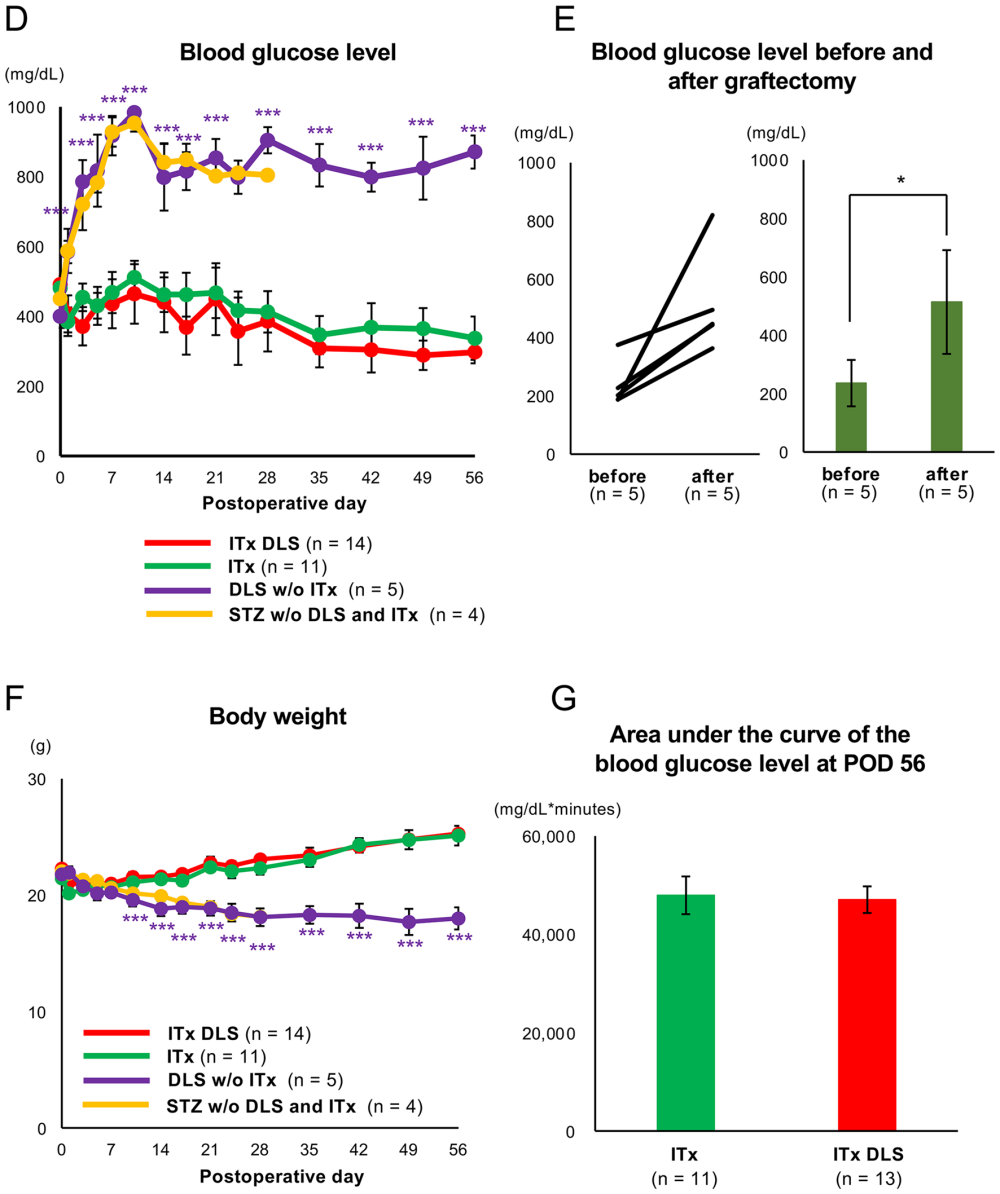
Therapeutic effect of the GHS-R1a antagonist in islet transplantation. (**A**) Scheme of DLS treatment protocol for islet transplantation (ITx DLS group). (**B**) Changes in plasma somatostatin level in the ITx DLS, ITx, DLS w/o ITx and STZ w/o DLS, and ITx groups. (**C**) Changes in plasma insulin level in the four groups. *Triangles* indicate the day of DLS injection. Data are presented as means ± SEMs. *P* value of < 0.05 was considered statistically significant. (**D**) and (**F**) Blood glucose (**D**) and body weight (**F**) levels after islet transplantation in ITx DLS (*red*; *n* = 14), ITx (*green*; *n* = 11), DLS w/o ITx (*n* = 5), and STZ w/o DLS and ITx (n = 4) groups. (**E**) Blood glucose levels before and after graftectomy in the ITx DLS group. (**G)** Glucose tolerance test (GTT) at POD 56. The data shows the area under the curve for the blood glucose level (AUC-GTT). Data are presented as means ± SEMs. *P* value < 0.05 was considered statistically significant.

### Limited contribution of GHS-R1a antagonist to the improvement of islet transplantation

The current study then assessed the therapeutic effects of DLS on islet transplantation using an animal model of diabetes. Mice with streptozotocin-induced diabetes received renal subcapsular islet transplantation, and following DLS intraperitoneal injections every 2 days for 15 days, mice were classified into the ITx DLS group (Fig. 4A). Accordingly, ITx DLS group had comparatively lower plasma somatostatin levels than the ITx group, which comprised islet-transplanted mice with no DLS treatment, on postoperative day (POD) 14 (0.89 ± 0.02 ng/mL in the ITx group vs. 0.59 ± 0.04 ng/mL in the ITx DLS group; *p* < 0.01; Fig. 4B). After discontinuing DLS administration, no difference was noted between both the groups (Fig. 4B). Moreover, the ITx DLS group maintained a higher plasma insulin level in until POD 28, although no significant differences was observed between both groups (208.20 ± 32.79 pg/mL in the ITx group vs. 360.40 ± 73.74 pg/mL in the ITx DLS group on POD 28; *p* = 0.09; Fig. 4C). However, the DLS w/o ITx and STZ w/o DLS and ITx groups maintained lower plasma insulin and somatostatin levels than the other two groups (Fig. 4B, C). These data indicated that DLS administration suppressed the release of somatostatin and may promote the release of insulin from transplanted islets. The therapeutic effects were abolished after DLS withdrawal.

**Figure 5:**
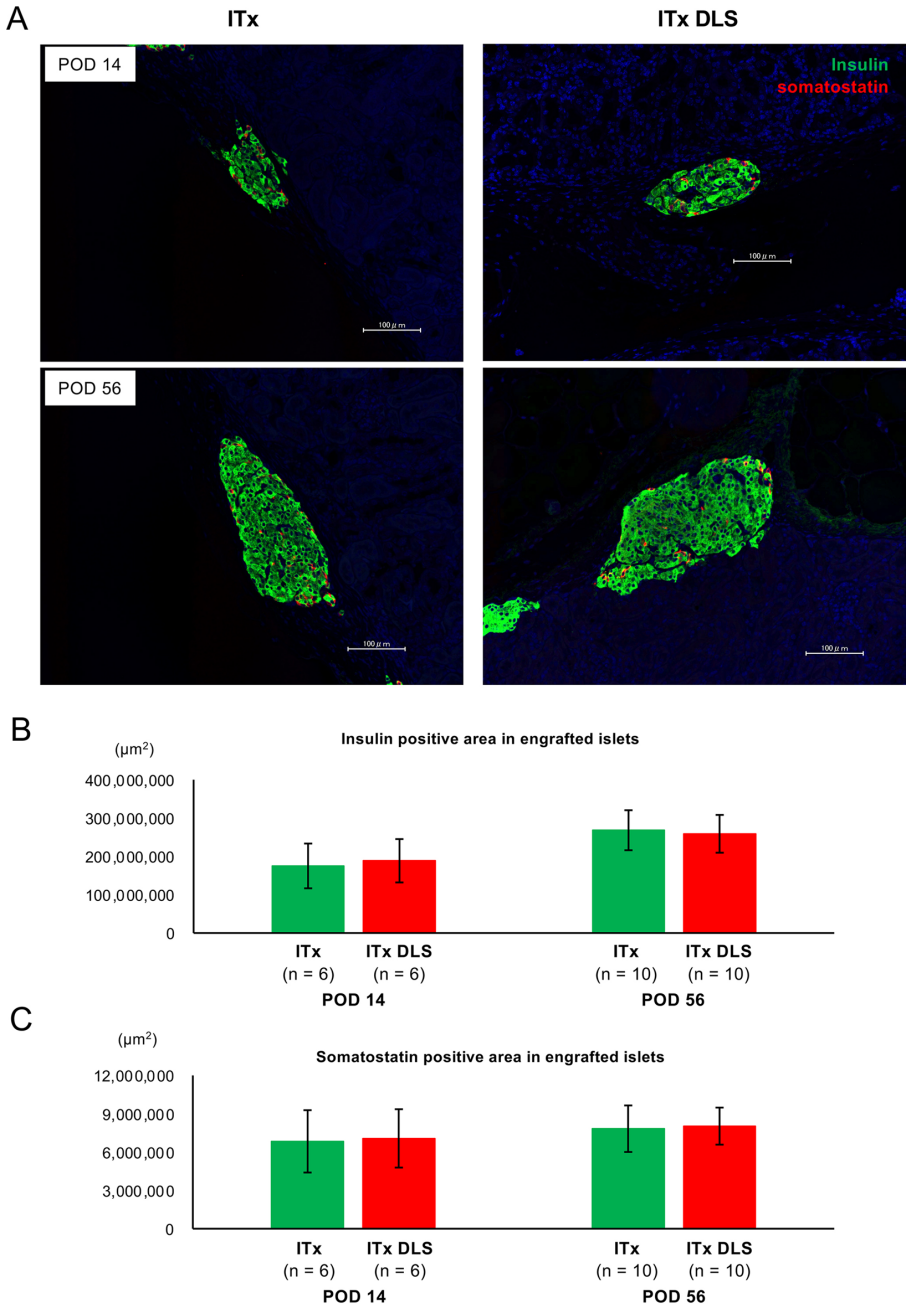
Histological findings of the graft in the ITx DLS and ITx groups. (**A**) Histological findings of transplanted islets in the renal subcapsular space of the ITx DLS and ITx groups at PODs 14 (*upper*) and 56 (*lower*) stained for insulin (*green*) and somatostatin (*red*). Nuclei were stained using DAPI (*blue*). (**B**) Insulin-positive area in engrafted islets. (**C**) Somatostatin-positive area in engrafted islets. Data are presented as means ± SEMs. *P* value < 0.05 was considered statistically significant.

Although DLS had clear therapeutic effects on transplanted islets, its impact on islet transplantation remained to be elucidated. Notably, mice that received DLS without islet transplantation showed no improvement in blood glucose levels (categorized as DLS w/o ITx group), indicating that DLS played no role in improving blood glucose level in animals with streptozotocin (STZ)-induced diabetes (Fig. 4D). The blood glucose level and body weight tended to subsequently improve in both the ITx DLS and ITx groups (Fig. 4D, F). No improvements in blood glucose and body weight were observed in the DLS w/o ITx and STZ w/o DLS and ITx groups. This suggests that DLS had no adverse effects (Fig. 4D, F). However, no difference between the two groups was noted throughout the observation period (56 days; Fig. 4D, F). Furthermore, the ITx DLS group did not show a superior area under the curve for the glucose tolerance test (AUC-GTT) on POD 56 (47,937.27 ± 3,835.97 mg/dL × min in the ITx group and 47,048.08 ± 2,706.24 mg/dL × min in the ITx DLS group; Fig. 4G). The transplanted islets in the ITx DLS group ameliorated blood glucose levels. Moreover, all the mice in the ITx DLS group exhibited re-elevation of blood glucose levels after graftectomy (289.20 ± 44.03 mg/dL before graftectomy vs. 532.70 ± 55.23 mg/dL; *p* = 0.021; Fig. 4E). However, the present study failed to demonstrate the supportive effects of DLS that contributed to the improved outcomes of islet transplantation. The therapeutic effect of DLS was also assessed according to different administration methods. Supplemental Fig. 5, which compares changes in the blood glucose level between mice with transplanted islets incubated with DLS (ITx DLS-incubated group) and the ITx DLS group, shows that the ITx DLS-incubated group was not superior but inferior. Figure 5 shows the histological images of the graft in the ITx DLS-IP and ITx groups on PODs 14 and 56. Transplanted islets were successfully engrafted in both groups (Fig. 5A). No differences in both insulin- and somatostatin-positive areas were noted between the ITx and ITx DLS groups (Fig. 5B and C), indicating that DLS did not affect the proliferation/diminishment of these cells.

## Discussion

GHS-R1a is a seven-transmembrane G protein-coupled receptor for acylated ghrelin expressed in various sites, such as the brain^18^, islets, thyroid, and heart^19^. Moreover, GHS-R1a does not bind to unacylated ghrelin considering that acylation by ghrelin O-acyl-transferase is an essential reaction for binding with this receptor^20,21^. Previous studies have revealed that ghrelin administration increased blood glucose levels in humans and rodents by decreasing insulin release from β cells^11,12^, indicating that ghrelin is the primary contributor to the decrease in insulin release. Furthermore, Dezaki et al. revealed that the binding of acylated ghrelin to GHS-R1a on β cells suppressed insulin release via cyclic AMP pathway suppression^13^. Moreover, DiGruccio et al. showed that acylated ghrelin acted on GHS-R1a found on δ cells and induced somatostatin secretion that restricted insulin release from β cells^15^. They also demonstrated that unacylated ghrelin did not increase the secretion of somatostatin^15^. Therefore, inhibiting the binding of acylated ghrelin to GHS-R1a enabled the estimation of the increase in insulin release by directly and indirectly acting on β cells via a decrease in somatostatin release from δ cells.

The current study demonstrated that the administration of specific GHS-R1a antagonist DLS promoted insulin expression/production and improved insulin-releasing function depending on change in glucose levels. DLS treatment also suppressed the expression/production of somatostatin, which may have improved the insulin-releasing function of β cells. Furthermore, continuous DLS administrations promoted a decrease in plasma somatostatin level. This phenomenon may have been induced by suppression of the production/release of somatostatin from δ cells by DLS, which contributed to the recovery of insulin release from β cells. The aforementioned data confirmed our hypothesis that preventing the binding of ghrelin to GHS-R1a directly and indirectly promoted insulin release from β cells. Additionally, data presented herein indicated that DLS did not affect cellular growth, which may improve islet function.

The in vitro assay showed that GHS-R1a antagonist administration could benefit islet transplantation. However, this treatment did not improve the therapeutic effect of islet transplantation in animals. The present study demonstrated that DLS administration every 2 days for 2 weeks may suppress the increase in plasma somatostatin level. The ITx DLS group maintained higher plasma insulin levels than the ITx group until POD 28. The decrease in plasma somatostatin and subsequent elevation of plasma insulin levels was abolished after stopping DLS administration. Moreover, although DLS may regulate somatostatin/insulin release by transplanted islets, it did not improve blood glucose levels, body weight, and glucose tolerance in a mouse model of diabetes that received islet transplantation.

Although GHS-R1a antagonist treatment may support the insulin-releasing function of transplanted islets, it may be insufficient to improve the therapeutic effects of islet transplantation considering that this treatment did not promote the proliferation of endocrine cells. Thus, some modifications to this strategy may be necessary for it to successfully affect islet transplantation. For instance, long-term continuous administration of GHS-R1a antagonist can be one such option. Another option could be the administration of a combination of acylated and unacylated ghrelin, which may be used as supportive therapy. Unacylated ghrelin has two useful roles in treating diabetes, namely, improving insulin sensitivity and cellular proliferation. Moreover, insulin sensitivity is defined as the ability of insulin to clear glucose from the blood. Glycogen and lipid syntheses by insulin are major contributors to insulin sensitivity^22^. Thus, improving insulin sensitivity can allow good management of blood glucose levels. Furthermore, unacylated ghrelin can increase islet cell mass and improve β cell survival. Granata et al. found that the administration of unacylated ghrelin increased the plasma insulin level, islet area, islet number, and β cell mass of animals with STZ-induced diabetes. Moreover, they revealed that unacylated ghrelin promoted the expression of pancreatic/duodenal homeobox-1 (Pdx-1), a promotor of pancreatic development, and BCL2, an antiapoptotic marker^23^, and exerted cytoprotective effects by preventing oxidative stress^24^. These effects may improve the therapeutic outcomes of islet transplantation using a GHS-R1a antagonist.

PP cells are endocrine cells that release pancreatic peptide. PP cells comprise a small proportion of islet cells, accounting for < 2% of the islet cells in humans and < 5% in rodents^25^. Recently, Fukaishi and colleagues elucidated that PP cells may get converted to β cells when β cells are impaired. They concluded that PP cells served as progenitors of β cells and contributed to the maintenance of homeostasis of pancreatic islets^26^. However, the correlation between PP and other endocrine cells has not been fully discussed. The current study confirmed that the percentage of GHS-R1a-positive PP cells was higher than those of β and δ cells. Although the correlation between PP cells and GHS-R1a has not been fully discussed for a long time, two recent publications have investigated such a correlation. After performing a comprehensive gene expression analysis using human and rodent islets, Yu and colleagues detected GHS-R1 gene expression in PP cells^27^. Furthermore, Gupta and colleague elucidated that GHS-R1a was expressed on most PP and δ cells in human and mouse islets^28^. Hence, GHS-R1a-positive PP cells might play a pivotal role in the regulation of glucose homeostasis. Further studies elucidating this correlation are recommended.

In conclusion, despite its limited effects, the GHS-R1a antagonist contributed toward improving the therapeutic effects of islet transplantation. Further studies are necessary to prove the efficacy of this treatment and establish strategies for improving islet transplantation.

## Materials and methods

### Ethical statement

All experimental protocols were approved by the Animal Care and Use Committee of Fukuoka University (approval number: 1907036). The handling of mice and experimental procedures was in compliance with the Principles of Laboratory Animal Care (Guide for the Care and Use of Laboratory Animals, National Institutes of Health publication 86-23, 1985).

### Animal

This study used 8- to 10-week-old C57BL/6 J male mice (CLEA Japan Inc., Tokyo, Japan) weighing 20–24 g as syngeneic donors for islet and diabetic recipients. They were housed under specific pathogen-free conditions and had free access to food and water. Fasting before a challenge or assessment was not undertaken except before GTT.

### Islet isolation

Donor mice received median laparotomy under general anesthesia using isoflurane (FUJIFILM Wako Pure Chemical Corporation, Osaka, Japan). Collagenase solution (Merck, Sigma-Aldrich, St. Louis, MO, USA) at 1 mg/mL was then infused into the pancreata via the common bile duct and distended. The pancreata was then digested using a water bath at 37 °C for 18 min. Islets were purified from the digestions by centrifugation at 2,000 rpm for 13 min using Biocoll separation solution (Biochrom, Berlin, Germany) at 1.100, 1.086, 1.077, and 1.040 density gradients by filtration using a 40 µm size Cell Strainer (Corning Inc., Corning, NY, USA) and handpicking. Purified islets were cultured overnight at 22 °C in low-glucose Dulbecco’s modified Eagle’s medium (L-DMEM; Thermo Fisher Scientific, Waltham, MA, USA) containing 10% fetal bovine serum (FBS; Thermo Fisher Scientific) and 1% penicillin–streptomycin (PS; Thermo Fisher Scientific).

### *Administration of GHS-R1a antagonist for *in vitro* examinations*

GHS-R1a antagonist, [D-Lys3]-GHRP-6 (DLS; R&D Systems), was administered to the isolated islets for in vitro examinations. Thereafter, 50 islet equivalents (IEQs: 1 IEQ means 150 µm-sized islets) were incubated with 4 µg/mL of DLS (defined as DLS group) or without DLS (no-treatment group) in L-DMEM with 10% FBS and 1% PS at 37 °C for 4 h.

### RT-PCR

Total RNA was extracted from 200 IEQs using TRIzol Reagents (Invitrogen, Thermo Fisher Scientific, Waltham, MA, USA) and the PureLink RNA Mini Kit (Thermo Fisher Scientific) according to the manufacturer’s instructions. RT was performed using the QuantiTect Reverse Transcription Kit (QIAGEN K.K., Tokyo, Japan).

RT-PCR analysis was performed using the CFX Connect Real-Time PCR Detection Systems (Bio-Rad Laboratories, Inc., Hercules, CA, USA) with the THUNDERBIRD SYBR qPCR Mix (Toyobo Co., LTD., Osaka, Japan). All primers were designed by Fasmac Co., Ltd. (Atsugi, Japan). The results were normalized to housekeeping genes (*Actb*). Data are presented as fold difference over the detectable Ct value, which was calculated using the ΔΔCt method. Primers for real-time RT-PCR are shown as follows:

**Table:** 

Primer	Sequence (5′–3′)	Tm (℃)
*Actb* forward	CATCCGTAAAGACCTCTATGCCAAC	67.2
*Actb* reverse	ATGGAGCCACCGATCCACA	69.0
*Ins2* forward	TCAAGCAGCACCTTTGTGGTT	63.1
*Ins2* reverse	TCCACCCAGCTCCAGTTGT	61.7
*Sst* forward	CTGGCTTTGGGCGGTGTC	69.2
*Sst* reverse	AAGTACTTGGCCAGTTCCTGTTTC	65.3
*Ghrl* forward	TCTGGGAAGAGGTCAAAGAGGC	67.7
*Ghrl* reverse	GGTAGGAGAGTGCTGGGAGTT	63.5
*Ghsr* forward	GACCAGAACCACAAACAGACAG	63.5
*Ghsr* reverse	GGCTCGAAAGACTTGGAAAA	63.1

### Extraction and measurement of protein from islets

Proteins (insulin, glucagon, and somatostatin) were extracted from 50 IEQs using 300 µL RIPA buffer (Sigma-Aldrich) containing protease and phosphatase inhibitor (Nacalai Tesque, Inc., Kyoto, Kyoto, Japan). Insulin content was measured using the enzyme-linked immunosorbent assay (ELISA) with the Mouse Insulin ELISA Kit (RTU) (FUJIFILM Wako Shibayagi Co., Shibukawa, Gumma, Japan). Somatostatin content was determined using the Somatostatin ELISA kit (Phoenix Pharmaceuticals, Inc., Burlingame, CA, USA). iMark Plate Reader (Bio-Rad Laboratories, Inc.) at OD450 with Microplate Manager Software (ver. 6.3, Bio-Rad Laboratories, Inc.) was used for reading sample absorbance.

### Glucose-stimulated insulin secretion assay

GSIS assay was performed on islets treated with and without DLS. During the preincubation process, 10 IEQs were incubated in low-glucose solution (3.3 mM) at 37℃ for 1 h. After washing, the islets were incubated in low-glucose solution at 37℃ for 1 h, and the supernatants were collected as samples. After incubation in high-glucose solution (16.5 mM) for 1 h, the supernatants were collected similar to that done with the low-glucose solution. The insulin concentration of the samples was quantified using the Mouse Insulin ELISA Kit (RTU; FUJIFILM Wako Shibayagi Co). The stimulation index, which was defined as the ratio of insulin content in samples from high- and low-glucose solutions, was calculated.

### Assessment of released somatostatin from islets

Ten IEQs were cultured in L-DMEM containing 10% FBS and 1% PS with 4 µg/mL of DLS (DLS) or without DLS (no treatment) at 37 °C for 4 h. After removing the islets, the somatostatin volume in the medium was measured using the ELISA kit as previously described following the manufacturer’s instruction.

### Assessment of viability of islets and islet residual rate

Islets after administration of the DLS (refer *Administration of GHS-R1a antagonist for *in vitro* examinations* section) or without treatment were cultured in L-DMEM containing 10% FBS and 1% PS for 7 days. Detection of live/dead cells in the islets at day 7 was performed by staining with Hoechst 33,342 and propidium iodide (PI; Thermo Fisher Scientific K.K.). The viability of the islets was defined as per the following formula: ([Hoechst 33,342-positive cells] − [PI-positive cells])/[Hoechst 33,342-positive cells] × 100 (%).

The islet residual rate was calculated using the percentage of residual numbers of cultured islets at days 3, 5, and 7 and compared with the numbers at the beginning of the culture.

### DM induction

Diabetes was induced in recipient mice by one-shot intravenous injection of STZ (180 mg/kg body weight; Merck, Sigma-Aldrich). Blood glucose levels in mice were measured using the Glutest Mint (Sanwa Kagaku Kenkyusho Co. Ltd., Nagoya, Japan) 4 days after injection. Mice with blood glucose levels exceeding 400 mg/dL were used for transplantation examination as recipients.

### Islet transplantation and treatment of GHS-R1a antagonist

Syngeneic 100 IEQs were transplanted into the renal subcapsular space of the left kidney of recipient mice. Thereafter, mice were classified into two groups according to DLS treatment. Mice with discontinuous intraperitoneal injection of 20 µg DLS at PODs 1, 3, 5, 7, 9, 11, 13, and 15 were classified into the ITx DLS group (Fig. 4A), whereas those without DLS treatment were classified into the ITx group. Furthermore, mice with transplantation of 100 IEQs incubated with 4 µg/mL DLS and those that received discontinuous DLS intraperitoneal injection at similar volumes and time without islet transplantation were prepared. The former was classified into the ITx DLS-incubated group, whereas the latter were classified into the DLS w/o ITx group. Mice with STZ-induced diabetes without DLS and islet transplantation were also prepared as negative control and classified into the STZ w/o DLS and ITx group.

### Assessment of the therapeutic effect of islet transplantation

The therapeutic effect of islet transplantation was assessed by monitoring blood glucose level, plasma insulin level, body weight, and changes in blood glucose level in the GTT at POD 56. Nonfasting blood glucose levels were measured in the morning of PODs 0, 1, 3, 5, 7, 10, 14, 17, 21, 24, 28, 35, 42, 49, and 56. Normoglycemia was defined as a blood glucose level of < 200 mg/dL. Blood samples for assessing plasma insulin levels were collected from the tail veins of mice on PODs 0, 3, 7, 14, 28, and 56. The collected blood volume was 200 µL. Samples were isolated in plasma by centrifugation at 16 g for 15 min and cryopreserved until measurement. Plasma insulin levels were measured using the Mouse Insulin ELISA Kit (RTU). Intraperitoneal glucose tolerance tests (IPGTTs) were also performed 2 months after transplantation. Glucose solution (2 g/kg body weight) was intraperitoneally injected after 10–12 h of fasting, and glucose levels were measured after 0, 30, 60, 90, and 120 min. The AUC-GTT was calculated. After finishing this monitoring, left nephrectomy was conducted for graftectomy. Blood glucose levels after graftectomy were measured and compared with those before the procedure (at POD 56).

### Histological assessment

The left kidneys recovered at PODs 14 or 56 and isolated islets embedded in 2% agarose gel (UltraPure LMP Agarose; Invitrogen) were fixed using 10% formalin neutral buffer solution and embedded in paraffin. Paraffin Sects. (3 μm) of specimens were subjected to immunohistochemistry to examine insulin (to detect β cells), somatostatin (to detect δ cells), pancreatic polypeptide (to detect PP cells), Ki-67 (for cellular proliferation), and GHS-R1a. The primary antibodies included guinea pig anti-insulin (1:2; Agilent, Dako, Tokyo, Japan), rabbit anti-somatostatin antibody (1:500; Abcam, Cambridge, UK), mouse anti-pancreatic polypeptide antibody (1:250; Immuno-Biological Laboratories Co., Ltd., Gunma, Japan), rabbit anti-Ki67 antibody (1:200; Abcam), and rabbit anti-GHSR antibody (1:500; Invitrogen). After incubation with the primary antibodies, Alexa 488-conjugated donkey anti-guinea pig (1:100; Abgent Cat#, 706–546-148, AB_2340473; Jackson Immunoresearch, West Grove, PA, USA), FITC-conjugated goat anti-mouse (1:100; Abgent Cat#, 115–095-072; Jackson Immunoresearch), and Cy3-conjugated goat anti-rabbit (1:100; Abgent Cat#, 111–166-047, AB_2338010; Jackson Immunoresearch) were used as secondary antibodies. Nuclear staining was performed using 4′,6-diamidino-2-phenylindole (DAPI; Invitrogen). All histological analyses were performed using a BZ-X700 microscope (Keyence, Itasca, IL, USA), after which findings were quantified for statistical evaluation. The expression rate of GHS-R1a on β/δ cells was calculated using the following formula: [GHS-R1a-positive β/δ cells] / [total β/δ cells in islet] × 100 (%). The Ki-67-positive rate was also quantified according to the percentage of Ki-67-positive cells per total islet cells. These quantifications were performed using the ImageJ® software (National Institutes of Health, Bethesda, MD, USA).

### Statistical assessment

Blood glucose level, plasma insulin level, body weight, and changes in blood glucose levels in the IPGTT were compared between the DLS-IP and other groups using a two-way repeated-measure analysis of variance. All multiple comparisons were assessed using Dunnett’s test. All data are presented as means ± standard errors of the mean (SEMs). Significant differences were defined as *p* < 0.05. All tests were two-sided, and all statistical analyses were performed using JMP12.0.0 (SAS Institute Inc., Cary, NC, USA).

### Statement on ARRIVE guidelines

Study was conducted in accordance with ARRIVE guidelines.

## Data availability

No datasets were generated or analyzed during the present study.

## Supplementary Information

Supplementary Information.

## References

[1] Hering BJ (2016). Phase 3 trial of transplantation of human islets in type 1 diabetes complicated by severe hypoglycemia. Diab. Care.

[2] Matsumoto S, Abalovich A, Wechsler C, Wynyard S, Elliott RB (2016). Clinical benefit of islet xenotransplantation for the treatment of type 1 diabetes. EBioMedicine.

[3] Shapiro AM (2000). Islet transplantation in seven patients with type 1 diabetes mellitus using a glucocorticoid-free immunosuppressive regimen. N. Engl. J. Med..

[4] Haller MJ (2018). Low-dose anti-thymocyte globulin (ATG) preserves beta-cell function and improves HbA1c in new-onset type 1 diabetes. Diab. Care.

[5] Matsuoka T (2020). Inhibition of NLRP3 inflammasome by MCC950 improves the metabolic outcome of islet transplantation by suppressing IL-1beta and islet cellular death. Sci. Rep..

[6] Yoshimatsu G (2017). Pancreatic beta-cell-derived IP-10/CXCL10 isletokine mediates early loss of graft function in islet cell transplantation. Diabetes.

[7] Hata T (2015). Nerve growth factor improves survival and function of transplanted islets via Trka-mediated beta cell proliferation and revascularization. Transplantation.

[8] Sakata N (2010). Hyperbaric oxygen therapy improves early posttransplant islet function. Pediatr. Diab..

[9] Saito T (2010). Islet transplantation using donors after cardiac death: report of the Japan Islet Transplantation Registry. Transplantation.

[10] Sakata N, Yoshimatsu G, Kodama S (2019). Development and characteristics of pancreatic epsilon cells. Int. J. Mol. Sci..

[11] Dezaki K (2004). Endogenous ghrelin in pancreatic islets restricts insulin release by attenuating Ca2+ signaling in beta-cells: implication in the glycemic control in rodents. Diabetes.

[12] Broglio F (2003). The endocrine response to ghrelin as a function of gender in humans in young and elderly subjects. J. Clin. Endocrinol. Metab..

[13] Dezaki K, Kakei M, Yada T (2007). Ghrelin uses Galphai2 and activates voltage-dependent K+ channels to attenuate glucose-induced Ca2+ signaling and insulin release in islet beta-cells: novel signal transduction of ghrelin. Diabetes.

[14] Arosio M (2003). Stimulatory effects of ghrelin on circulating somatostatin and pancreatic polypeptide levels. J. Clin. Endocrinol. Metab..

[15] DiGruccio MR (2016). Comprehensive alpha, beta and delta cell transcriptomes reveal that ghrelin selectively activates delta cells and promotes somatostatin release from pancreatic islets. Mol. Metab..

[16] Park S, Jiang H, Zhang H, Smith RG (2012). Modification of ghrelin receptor signaling by somatostatin receptor-5 regulates insulin release. Proc. Natl. Acad. Sci. USA.

[17] Delhanty PJ (2010). Unacylated ghrelin rapidly modulates lipogenic and insulin signaling pathway gene expression in metabolically active tissues of GHSR deleted mice. PLoS ONE.

[18] Albarran-Zeckler RG, Smith RG (2013). The ghrelin receptors (GHS-R1a and GHS-R1b). Endocr. Dev..

[19] Poher AL, Tschop MH, Muller TD (2018). Ghrelin regulation of glucose metabolism. Peptides.

[20] Gutierrez JA (2008). Ghrelin octanoylation mediated by an orphan lipid transferase. Proc. Natl. Acad. Sci. USA.

[21] Yang J, Brown MS, Liang G, Grishin NV, Goldstein JL (2008). Identification of the acyltransferase that octanoylates ghrelin, an appetite-stimulating peptide hormone. Cell.

[22] Angelidi AM, Filippaios A, Mantzoros CS (2021). Severe insulin resistance syndromes. J. Clin. Invest..

[23] Granata R (2010). Unacylated ghrelin and obestatin increase islet cell mass and prevent diabetes in streptozotocin-treated newborn rats. J. Mol. Endocrinol..

[24] Granata R (2012). Des-acyl ghrelin fragments and analogues promote survival of pancreatic beta-cells and human pancreatic islets and prevent diabetes in streptozotocin-treated rats. J. Med. Chem..

[25] Brereton MF, Vergari E, Zhang Q, Clark A (2015). Alpha-, delta- and PP-cells: are they the architectural cornerstones of islet structure and co-ordination?. J. Histochem. Cytochem..

[26] Fukaishi T (2021). Characterisation of Ppy-lineage cells clarifies the functional heterogeneity of pancreatic beta cells in mice. Diabetologia.

[27] Yu XX (2021). Sequential progenitor states mark the generation of pancreatic endocrine lineages in mice and humans. Cell Res.

[28] Gupta D (2021). High coexpression of the ghrelin and LEAP2 receptor GHSR with pancreatic polypeptide in mouse and human islets. Endocrinology.

