# Supplementary material for: Therapeutic effects of acylated ghrelin-specific receptor GHS-R1a antagonist in islet transplantation
Source: Sci Rep. 2021 Oct 28;11:21239. doi: 10.1038/s41598-021-00740-6 (PMC8553779; doi:10.1038/s41598-021-00740-6)
Supplement: Supplementary file 1 — Supplementary Information. [file 41598_2021_740_MOESM1_ESM.pdf]

Supplemental Figure 1. Determination of the proper concentration of DLS for this study

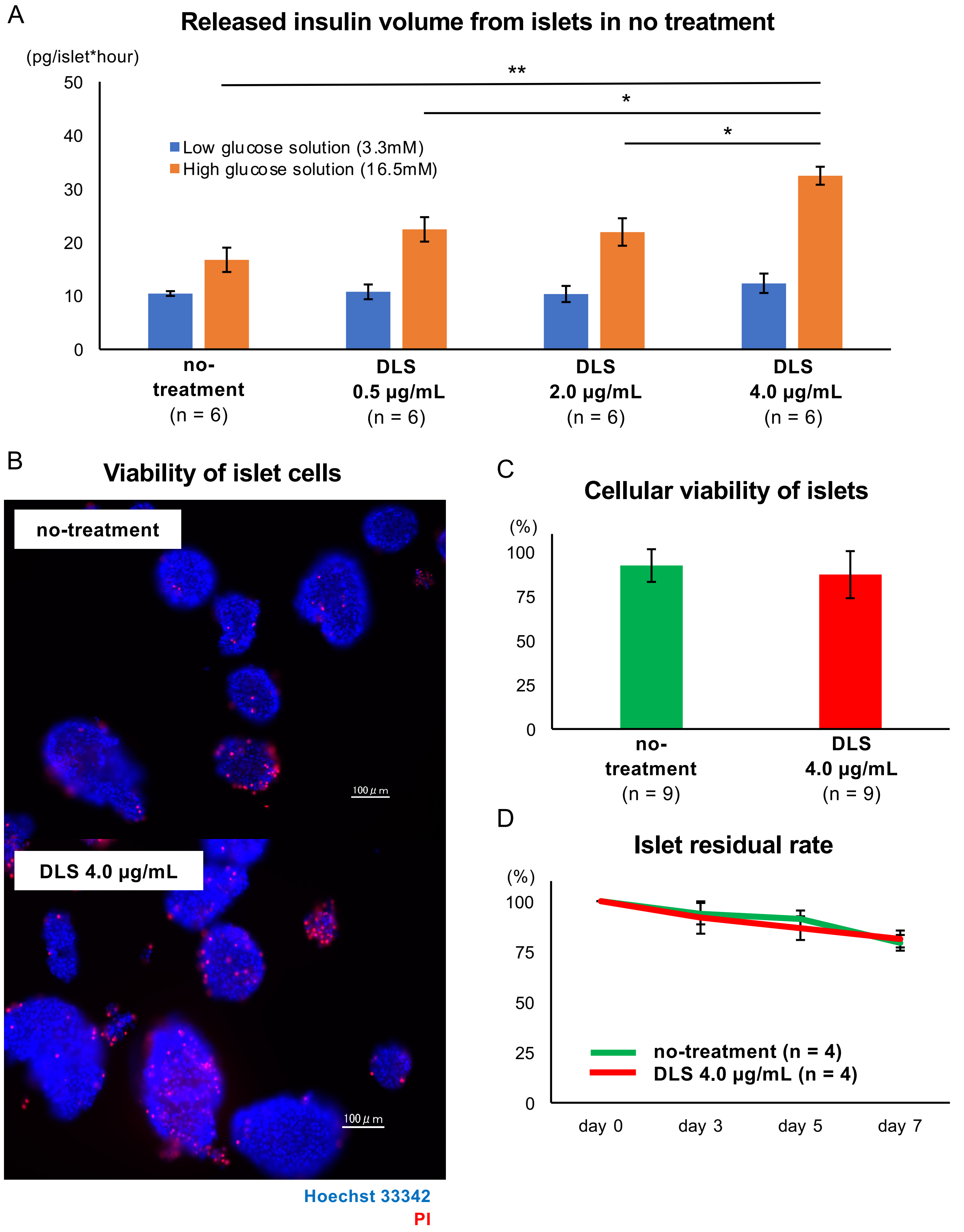

A Glucose-stimulated insulin secretion: released insulin volume from islets in the no-treatment, DLS 0.5 μg/mL, DLS 2.0 μg/mL, and DLS 4.0 μg/mL groups under low (*blue*)-/high (*orange*)-glucose stimulation. **B** Staining to evaluate the viability of islet cells at day 7. Hoechst 33342 (*blue*) was used for detecting total islet cells and propidium iodide (PI; *red*) for dead islet cells. *Scale bar* 100 μm. **C** Cellular viability of islets at day 7. **D** Islet residual rate until day 7 after the beginning of the culture. The percentage of residual numbers of cultured islets was shown and compared with the numbers at the beginning of the culture. Data are presented as means ± SEMs. *P* value < 0.05 was considered statistically significant. **p* < 0.05 and ***p* < 0.01.

Supplemental Figure 2. Ki-67 expression in islet cells

A

Islets stained for insulin and Ki-67

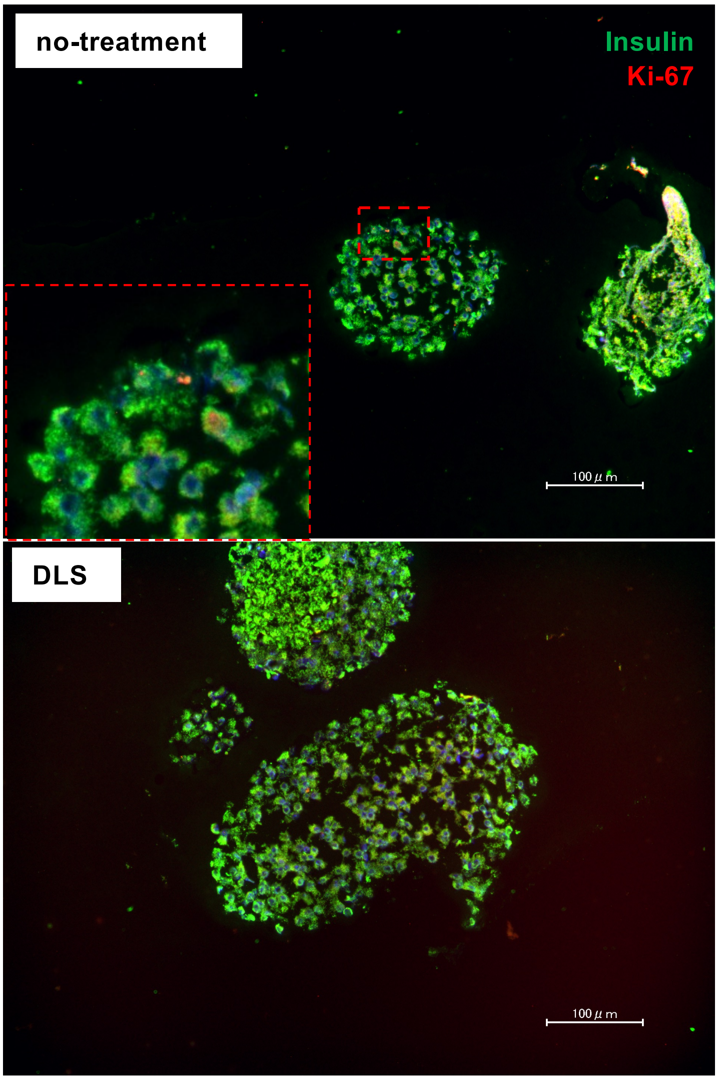

B

Ki-67-positive rate

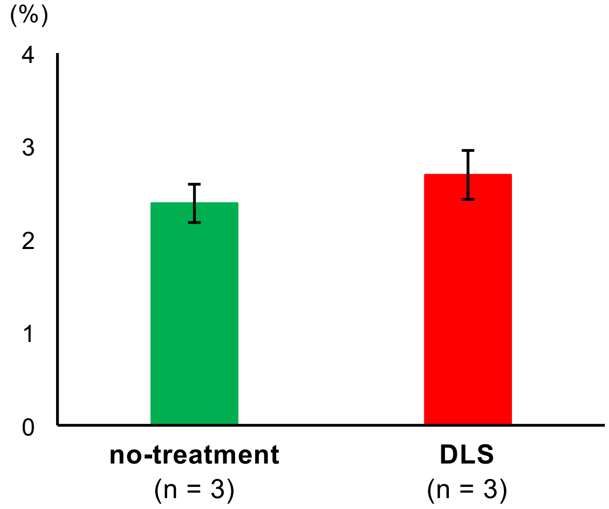

A Isolated islets with or without DLS treatment stained for insulin (green) and Ki-67 (*red*). Nuclei were stained using DAPI (*blue*). **B** Ratio between Ki-67-positive islet cells and total cells in the no-treatment and DLS groups. Data are presented as means ± SEMs. *P* value < 0.05 was considered statistically significant.

Supplemental Figure 3. Histological findings of islets stained for GHS-R1a

A

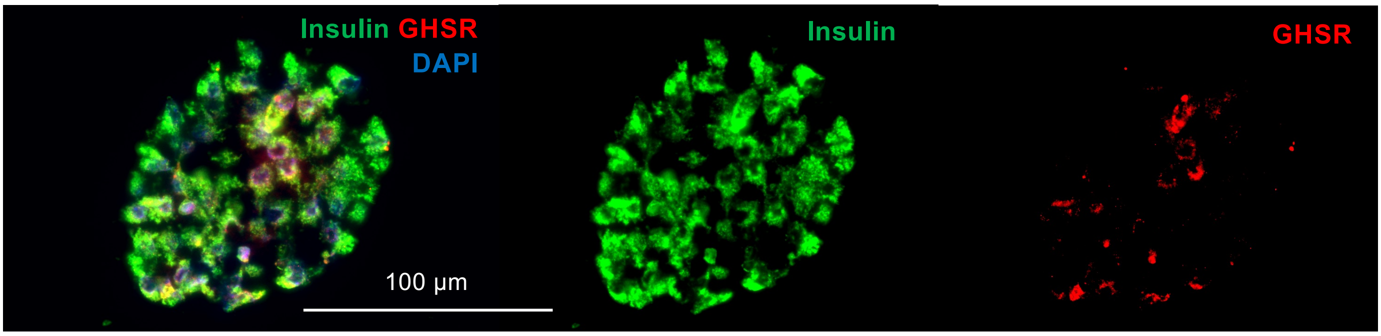

B

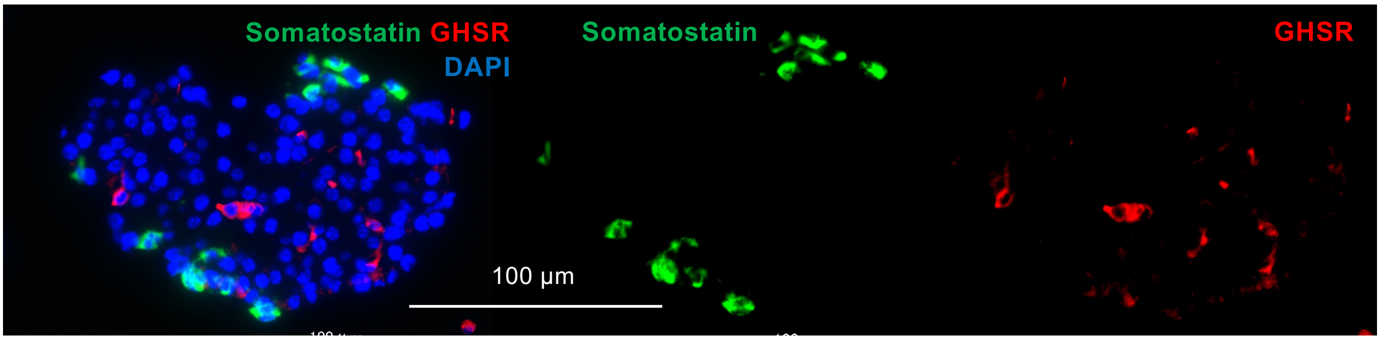

C

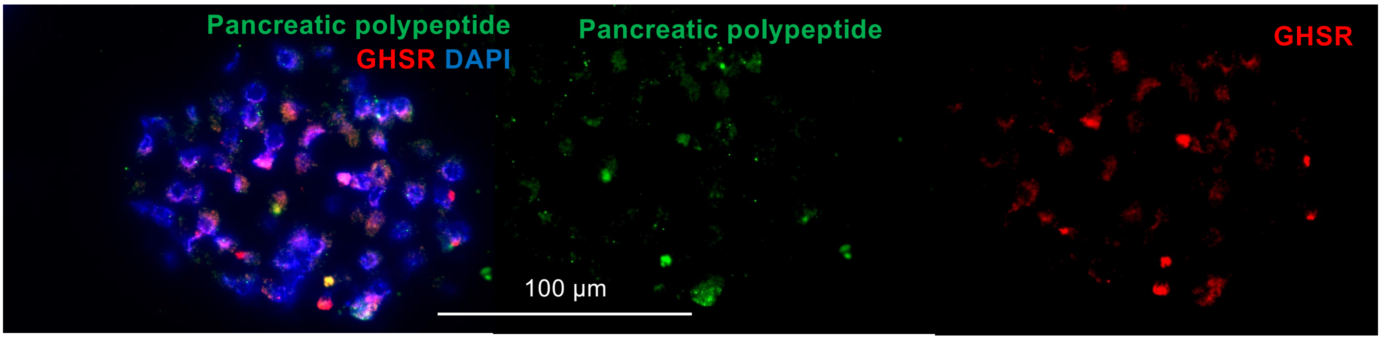

DLS-treated islets were stained for insulin (*green*: **A**), somatostatin (*green*: **B**), pancreatic polypeptide (*green*: **C**), and GHS-R1a (*red*). Nuclei were stained using DAPI (*blue*). *Left*: merged image; *Center*: insulin stainingimage; *Right*: GHSR stainingimage. The size of the scale bar is 100 µm.

Supplemental Figure 4. Expression of *Gca* and *Ghrl* in islets

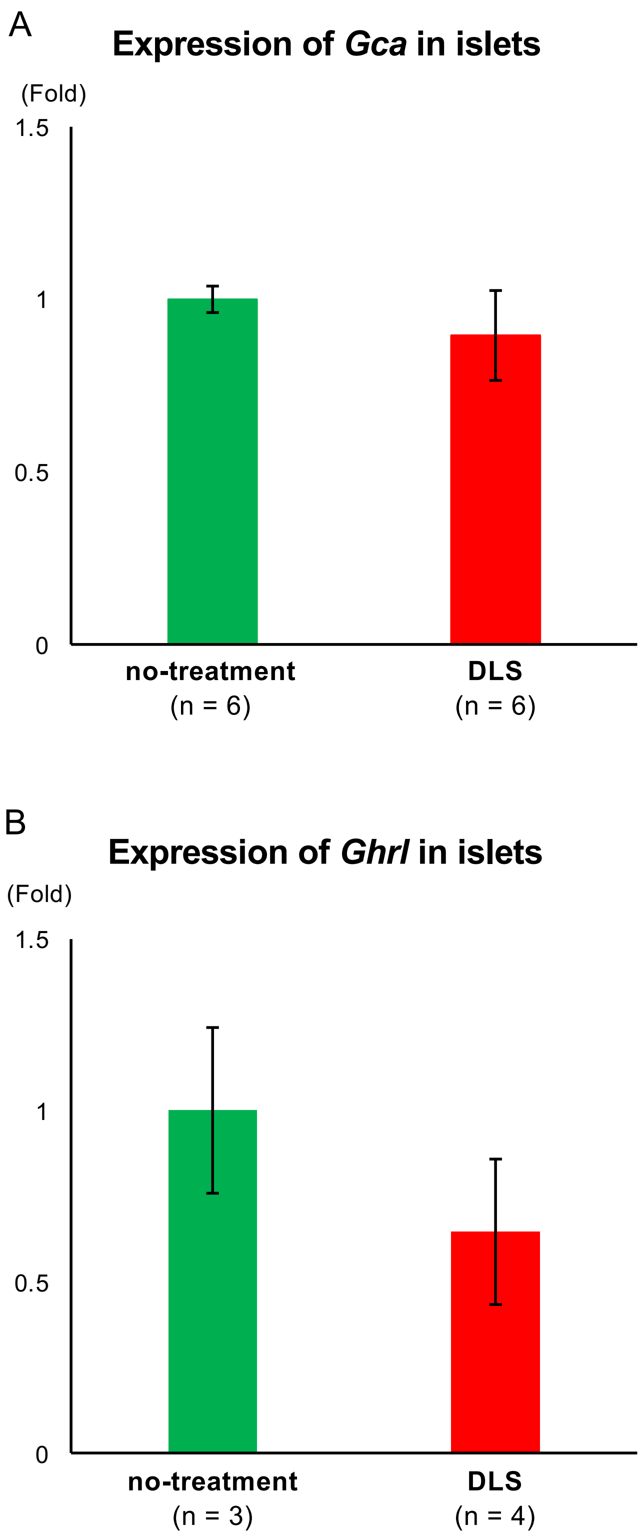

Expression of *Gca* (**A**) and *Ghrl* (**B**) in the islets of the no-treatment and DLS groups. Gene expression was quantified using real-time RT-PCR with *Actb* as the internal control. Data are presented as means ± SEMs. P value < 0.05 was considered statistically significant.

Supplemental Figure 5. Change in blood glucose levels after islet transplantation in the ITx DLS and ITx DLS-incubated groups

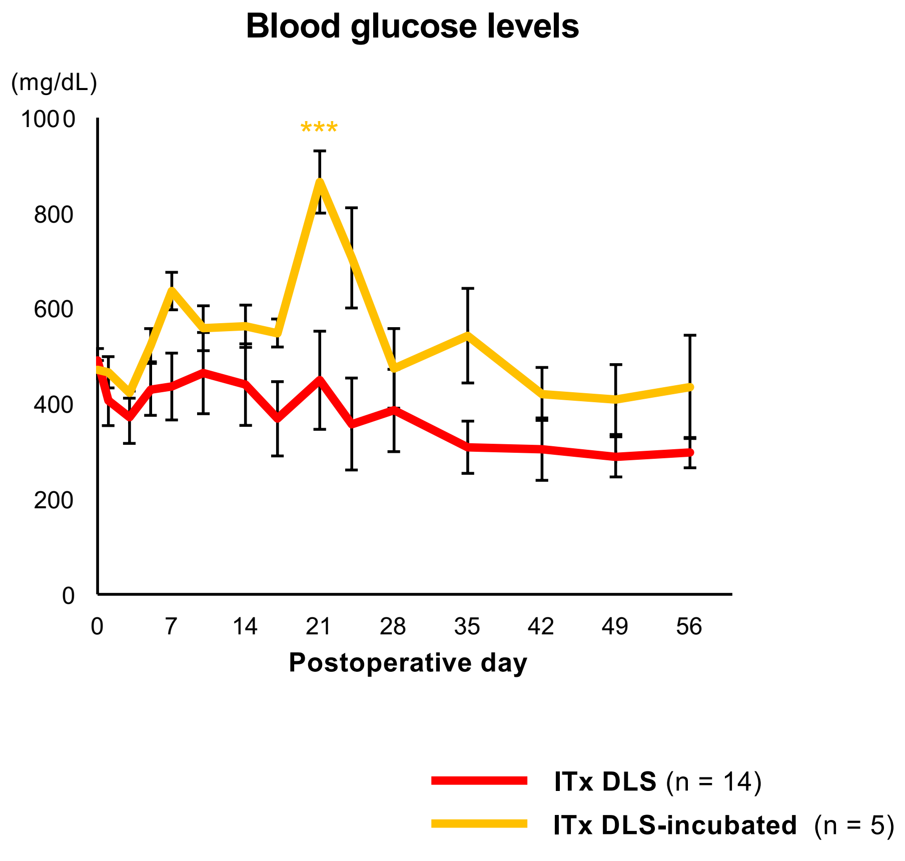

Data are presented as means ± SEMs. *P* value < 0.05 was considered statistically significant.
